# Establishing a Successful Bioinformatics Core Facility Team

**DOI:** 10.1371/journal.pcbi.1000368

**Published:** 2009-06-26

**Authors:** Fran Lewitter, Michael Rebhan

**Affiliations:** 1 Bioinformatics and Research Computing, Whitehead Institute for Biomedical Research, Cambridge, Massachusetts, United States of America; 2 Novartis Institutes for BioMedical Research, Basel, Switzerland; University of California San Diego, United States of America

There are many issues to consider when establishing and building a Bioinformatics Core Facility. Mission, funding, scope of projects, organizational context, infrastructure, software support, and training are among the many to consider. One of the most important and arguably the most critical to success is building the team to execute the mission of the group. We devote this Perspective to our thoughts on this and related issues and how they affect the mid- to long-term success of the Core Facility, in particular its ability to make strong scientific contributions.

Although Bioinformatics Core Facilities come in many different variants, depending on their history, mission, and institutional environment, they all share one challenge during the initial and consolidation phase: to recruit and retain the right people who thrive in such roles. One of the key requirements is that team members are able to learn new methodologies and expand into new areas as needed by the evolution of research methods. Bioinformaticians with sufficient and diverse experience are a relatively rare species. Therefore, hiring in the initial phase of a Core Facility's development often has to focus on bioinformaticians with experience in at least some of the key areas the group is expected to work in. A more mature and sustainable setup is often characterized by at least two to three senior staff with diverse experience but different focus areas, plus more junior ones in different stages (students, postdocs, and new staff). Such units are then able to offer the expertise required to competitively address difficult problems at the forefront of science.

In environments that expect considerable scientific contributions from the Bioinformatics Core Facility, one of the major success factors is to have people on the team who have extensive formal background in biology plus several years of experience in applying bioinformatics methods to biological problems. Many projects will require considerable biological insight in addition to hands-on experience with bioinformatics tools and scripting (in languages such as Perl, R, etc.), and at least basic knowledge of statistics and experimental design. In addition to those “in silico biologists”, some projects will require people with more extensive background in areas that biologists often cannot cover well, such as data mining and analysis, computer science, structural biology and biochemistry, more advanced statistics and experimental design, software engineering, theoretical biology, and so on, depending on the institution and the mission of the Facility. In such mature Facilities, staying up to date with the most relevant new approaches becomes attainable, as different experts can efficiently share ideas taken from the literature and from scientific meetings.

Achieving a high level of quality in providing scientific and technical solutions depends on several factors that include: a) allowing the bioinformaticians to spend 20%–40% of their time to develop mid-term focus areas that combine certain types of biological questions with related bioinformatics approaches; b) encouraging regular discussion on best practice within the unit, in particular the pros and cons of different approaches, and related resources; c) careful selection of the most relevant datasets and methods for a given problem (which can take some time if there isn't sufficient overlap with previous projects); d) designing solutions that, if possible, combine independent lines of evidence to make results as reliable and informative as possible; e) meaningful communication with the experimentalists on the scientific goals and their context (in many cases the formulation of the original request is the starting point of a discussion that results in solutions that address the main underlying problems more effectively), and what can be expected from the Facility (to avoid disappointments due to unrealistic expectations, which can be a major problem); and f) communicating the results to the experimentalist in a way that works for the target audience (often requiring many iterations of analyses and lab work).

As a measure of the maturity of the Bioinformatics Core Facility, not only will the contributions be valued by at least the early adopters in the institution, but the latter may then start to request input from bioinformatics experts in data-intensive projects that are designed as true interdisciplinary team efforts from the outset. For many bioinformaticians, this is where they always wanted to be. An early involvement of experienced bioinformaticians will usually have positive effects on the quality and relevance of the experimental design, and on the relevance of the produced data for the bioinformatics approaches that are needed to answer the scientific questions at hand.

Many Bioinformatics Core Facilities, however, do not reach that mature stage, and are caught, in extreme cases, in a “firefighting mode”, a vicious cycle between highly diverse, mostly urgent and hardly prioritized requests, and insufficient resources for developing high-quality solutions that make significant contributions to the output of the institution. Not surprisingly, it is notoriously difficult to attract and retain good people in such environments. Unfortunately, the problem is sometimes not analyzed systematically and recognized as such by the stakeholders. In such situations, open communication about the challenges and formalization of the communication to the main stakeholders in the form of a Core Facility committee can be a solution, if some understanding of the resources required to adequately address problems has developed.

The first two to three years are crucial, therefore, in many cases for building up the unit, in partnership with at least a few scientists who have sufficient interest and vision, and a strategic interest in taking advantage of bioinformatics capabilities in their research. Once the success stories are ready for presentation and for other forms of communication, this can help to extend institutional support for developing the Core Facility into the mature stage described above, which provides additional resources for supporting a larger group of customers. In our experience, which includes discussions with other managers of Bioinformatics Core Facilities within the “bioinfo-core” community (http://www.bioinfo-core.org), the following factors can be crucial for overcoming the often-difficult first years.

1. Developing the mission objectives of the Core Facility: does it include or exclude scientific collaborations (and co-authorship as an evaluation criterion), application development and hosting, statistics and experimental design support, educational campaigns, taking care of hardware, development of innovative bioinformatics methods, and so on? Is there an understanding of the resources needed to address those problems adequately?
2. Deciding on the right mix of people to be hired for this environment, after getting to know the early adopters and the institution to a degree that allows the selection of the most relevant skill sets.
3. Developing a good balance between small projects with a quick turnaround and larger collaborations that require in-depth literature study, exploration of alternative approaches, and extensive discussion (the latter are crucial for developing good showcases and strategic partnerships, and usually result in co-authorship on publications; small and swift projects, on the other hand, can help to build up partnerships and mutual understanding).
4. Finding strategies for addressing problems quickly and effectively (and related best practice, see above). This often includes reusable tools and datasets and the development of a dedicated bioinformatics computing environment that allows quick prototyping of alternative solutions to problems.
5. Transparent prioritization and careful time management helps in dealing with demand overload. This usually requires regular communication with partners and stakeholders, and helps to avoid situations in which staff become too scattered in terms of the different types of ongoing projects at a particular time. Proper management of the above challenges will have considerable impact on the quality of the solutions and often also affects the retention of experienced staff.
6. The head of the Bioinformatics Core Facility needs to make sure that those who request help from bioinformatics experts are comfortable with the process and the people (a certain degree of informality and getting-to-know-each-other can be very helpful here), while also balancing this goal of responsiveness and customer focus with the ability to manage expectations and urgent requests during times of high demand to provide an environment that allows sufficient time for quality work and capability development. This sometimes also includes the management of situations in which the bioinformaticians are going off track.
7. Staying connected: keeping in touch with new trends in the research strategy of individual investigators, understanding what is most relevant, and regularly collecting feedback on the relevance of the Core Facility.

## Staying on Top of New Developments

To stay up to date with new developments in the numerous fields related to bioinformatics, and to develop the skill set in the group, sufficient time needs to be reserved for keeping up with the literature, and for testing new approaches that are likely to be relevant for the mission of the Core Facility. In times of strong demand, this can become close to impossible. If this condition lasts long, the ability of the unit to deal with cutting-edge science will decrease over time. Of course, regular attendance at seminars, symposia, and larger meetings is important in this context as well, to allow exchange on best practice and bioinformatics approaches, in particular on experimental approaches for new data-intensive technologies (such as deep sequencing, see the Perspective on Managing and Analyzing Next-Generation Sequence Data in this issue, doi: 10.1371/journal.pcbi.1000369).

## Building a Local Bioinformatics Community

In addition to the staff affiliated with the Bioinformatics Core Facility, those who are part of other groups but in some sense also part of an informal local bioinformatics community can play an important role. They can include students focused on bioinformatics aspects of a project, technical experts, and scientists who see bioinformatics as an integral part of their strategy. Journal clubs and other events can help to build lively communities, and to foster quality, reusability, and open debate. For members, being part of such a community can be vital for the quality of their work, for accessing resources, and for guidance. For the Facility, they can provide a valuable resource, may function as a bridge into their units, and help with communication challenges.

## Outreach

A topic not yet discussed is that of outreach. How can a Bioinformatics Core Facility make a broader impact on the community, and how can the core team benefit? There are numerous examples of activities that fall under the category of outreach, some of which are mentioned here. Some successful examples include offering bioinformatics courses to biologists in the greater scientific community, presenting talks and other events to non-scientists on various topics in bioinformatics, providing opportunities within the core to mentor people training in bioinformatics, networking with other local or regional Bioinformatics Core Facilities, and building tools for the broader scientific community.

These activities have been successful for multiple reasons. The team gets the opportunity to share knowledge and excitement about our field with others who may or may not otherwise have the opportunity to be exposed to this material. It also builds confidence and relevance to the team by them having to prepare materials for people with limited or no knowledge about bioinformatics. Furthermore, the networking aspect can be invaluable for sharing information and experiences. Sharing of tools helps to put more emphasis on the quality and usability of software we build.

## Relevance for Stakeholders and Advocates

For the biologists who consider themselves stakeholders in a planned or existing Bioinformatics Core Facility, we recommend using this Perspective as a starting point for discussions with the head of the Bioinformatics unit on Core Facility setup and consolidation. Situations will vary and will most certainly require customized solutions that take into account local conditions, but we hope that the lessons learned above will provide useful guidance in this process. With the complexity of related data-intensive scientific questions steadily increasing, mostly due to technological developments, institutions that understand how to handle this interdisciplinary challenge are increasingly at an advantage. Institutions such as those who presented at the ISMB 2008 meeting (see the slides at http://www.bioinfo-core.org/index.php/ISMB_2008:_BoF_on_best_practices_in_running_bioinformatics_cores) may provide reference examples. In institutions where such a Core Facility is missing or in early stages, collaborations with bioinformatics or computer science research groups can help to address some of the problems and to collect some more experience, but they will fall short of dealing effectively with areas that are not within the narrow research scope of the collaborators.

